# Documented patient consent for ambient video monitoring technologies in an academic hospital: a cross-sectional analysis of consent outcomes and demographic differences

**DOI:** 10.3389/fdgth.2026.1969218

**Published:** 2026-10-21

**Authors:** Emily L. Godsey, Taylor M. Galloway, Jennifer S. Crews, Scott A. Helgeson

**Affiliations:** 1 Department of Ambient Intelligence, Mayo Clinic, Jacksonville, FL, United States; 2 Department of Innovation, Mayo Clinic, Jacksonville, FL, United States; 3 Digital Transformation, Mayo Clinic, Jacksonville, FL, United States; 4 Department of Nursing, Mayo Clinic, Jacksonville, FL, United States; 5 Division of Pulmonary Medicine, Department of Medicine, Mayo Clinic, Jacksonville, FL, United States; 6 Department of Critical Care Medicine, Mayo Clinic, Jacksonville, FL, United States

**Keywords:** ambient intelligence, artificial intelligence, digital health, health equity, patient consent, video monitoring

## Abstract

**Objective:**

To quantify the documented consent outcomes for ambient monitoring technologies in an academic hospital and evaluate whether documented consent acceptance differed by age, gender, and race.

**Patients and methods:**

We performed a retrospective cross-sectional study of 9,522 consecutive patients approached for ambient monitoring consent during routine hospital care from 1/1/2025 to 2/12/2026. Consent status was categorized as received, refused, or missing/unable. The primary multivariable analysis used a binary outcome of documented acceptance (received vs. refused or missing/unable) modeled with age, gender, and race. Secondary models used age categories.

**Results:**

Overall, 9,125 patients (95.8%) consented, 184 (1.9%) refused, and 213 (2.2%) had missing or unobtainable consent. Acceptance did not differ by gender (95.6% among female patients vs. 96.1% among male patients; adjusted odds ratio [aOR] 1.11, 95% CI 0.91–1.36; *p* = 0.30). Compared with White patients, lower adjusted odds of acceptance were observed among Black or African American patients (aOR 0.66, 95% CI 0.49–0.88; *p* = 0.005) and patients categorized as Unknown/Declined/Other (aOR 0.56, 95% CI 0.37–0.83; *p* = 0.004). Acceptance was highest among Asian patients, but the adjusted difference was not significant (aOR 2.44, 95% CI 0.90–6.59; *p* = 0.079). Acceptance exceeded 95% across all age groups and was highest among patients aged 65–74 years (96.7%). Younger patients more often refused, whereas older patients more often had missing or unobtainable consent.

**Conclusion:**

Documented consent acceptance was high in this single academic hospital. Acceptance did not vary by gender, but modest differences were observed by race and age-related patterns. Consent status alone does not establish patient comprehension, voluntariness, motivation, or durable endorsement. These findings demonstrate operational feasibility while underscoring the need for transparent consent, subgroup monitoring, and prospective evaluation of patient experiences.

## 1 Introduction

Artificial intelligence (AI), ambient intelligence, and passive sensing technologies are becoming more common in hospitals to support clinical surveillance, patient safety, and digital transformation of care delivery (1–4). In parallel, video-enabled monitoring systems have expanded across inpatient settings for virtual observation, remote clinical support, and contactless physiologic or behavioral assessment, with adoption accelerating during the COVID-19 pandemic (4–9). Although these technologies offer opportunities for earlier detection of clinical deterioration and more continuous monitoring, their real-world implementation depends not only on technical performance but also on patient acceptance (4, 5, 10–12).

Patient acceptance is a prerequisite for successful integration of AI-enabled systems in hospital care (8, 11–13). While patients and the public often express cautious optimism toward AI when it is framed as augmenting human care, concerns about privacy, surveillance, data security, bias, and loss of autonomy remain prominent (11, 12, 14–19). These concerns are only amplified for ambient video technologies, which involve continuous or near-continuous visual data capture in highly personal inpatient environments (5–7, 9). Prior studies suggest that acceptance is shaped by how monitoring is explained, how consent is obtained, the visibility of privacy protections, and trust in the institution deploying the technology (5–7, 9, 14–17, 19).

Acceptance of digital health technologies may also vary across demographic groups. Research on telemedicine and other patient-facing technologies has demonstrated disparities by age, race and ethnicity, and social context, alongside broader concerns about trust and equity in healthcare systems (20–32). Growing evidence that AI systems can reproduce or exacerbate existing inequities has intensified the need to understand acceptance patterns across populations (33–35). Despite rapid institutional investment in ambient clinical monitors, data on patient consent and acceptance of ambient video monitoring in routine inpatient practice remain limited (5–13).

We conducted a study of operational consent records for ambient video monitoring in a large academic hospital cohort to quantify documented consent outcomes and examine whether documented acceptance differed by age, gender, and race. We hypothesized that overall documented acceptance would be high, but that measurable subgroup differences would be observed.

## 2 Materials and methods

This was a retrospective cross-sectional study performed at a tertiary academic medical center in the Southeast USA from 1/1/2025 to 2/12/2026. This study was deemed exempt by the institutional review board (#26-001853). All patients who were admitted to the hospital during the study period were included. There were no exclusion criteria.

All patients admitted to our hospital are approached for consent to ambient monitoring technologies as part of routine hospital operations. Consent was obtained using a standardized electronic or paper acknowledgment. The acknowledgement described the use of camera- and sensor-based technologies located within the patient room, including audio-video monitoring capabilities intended to support patient safety, detect environmental risks, monitor movement, and enable remote clinical care.

It stated that collected data could be used for clinical care, healthcare operations, education, and technology development, including de-identified use, internally or by external partners to improve or develop technologies. It also stated that participation was optional, declining would not affect care, consent could be revoked at any time, and collected data were not incorporated into the legal medical record. Consent applied to the current hospitalization and future hospitalizations unless revoked.

Consent was integrated into routine inpatient and emergency department workflows and typically initiated during hospital or emergency department registration. Patients were presented with the electronic consent form at admission, and consent status was recorded in the electronic health record. Activation and deactivation of ambient monitoring technologies were automated based on consent status through integration between the electronic health record and the monitoring system. Consent status updates triggered system-level changes such that monitoring capabilities were enabled when consent was documented and disabled when consent was declined, not completed, or revoked.

This automated approach minimized reliance on manual processes, reduced the risk of inadvertent activation in patients without documented consent, and aligned system activation with the consent status recorded in the electronic health record.

Patients could revoke consent at any time during or after hospitalization through multiple pathways, including direct communication with care teams or completion of a revocation form. Revocation triggered both documentation updates within the electronic health record and immediate deactivation of monitoring technologies in the patient room. Patients were provided with standardized educational materials at registration and inpatient units to explain the purpose of the technology, privacy protections, and common questions. These materials were iteratively refined based on patient feedback, including input from institutional patient advisory groups.

Staff received training on consent workflows, documentation processes, and procedures for obtaining and revoking consent. In-room indicators signaled when ambient monitoring technologies were active, providing additional transparency during hospitalization. Consent status was recorded as routine operational documentation within the electronic health record and extracted for analysis.

The primary descriptive outcome was operational consent status for ambient monitoring technologies. Three analytic categories were created: received, refused, and missing/unable. “Received” represented records with documented consent. “Refused” included records coded as patient refused and revoked, reflecting active nonacceptance. “Missing/unable” included missing consent, unable to obtain, and not received, reflecting passive or incomplete consent capture. This grouping preserved the operational distinction between active refusal and the absence of completed consent.

Descriptive statistics summarized the cohort overall and by consent outcome. Categorical variables are reported as counts and percentages. Age was summarized continuously and categorically using groups of 18–44, 45–64, 65–74, and ≥75 years. Continuous age is reported as mean and standard deviation where applicable.

Bivariable analyses compared consent outcomes across demographic groups. Chi-square tests of independence evaluated associations of gender, race, and age group with the three-level consent outcome. Separate chi-square tests evaluated the binary outcome of documented acceptance (received, refused, or missing/unable). Continuous age was compared across the three consent categories using one-way analysis of variance and confirmed using the Kruskal–Wallis test. For the binary acceptance outcome, continuous age was compared using the Mann–Whitney U test.

To identify factors independently associated with documented acceptance, binary multivariable logistic regression was performed with received consent as the dependent category and age, gender, and race as covariates. Odds ratios (ORs) with 95% confidence intervals (CIs) were estimated. Female gender and white race were reference categories and low-frequency race responses were grouped as Unknown/Declined/Other category. The primary model treated age as a continuous variable. A secondary model replaced continuous age with age categories using 65–74 years as the reference, to assess nonlinearity in age-related patterns. Sensitivity analyses assessed robustness to alternative classifications of low-frequency consent statuses. These analyses reclassified revoked consent as accepted, excluded revoked cases, and repeated the regression among patients with an explicit yes/no decision after excluding missing or unobtainable consent categories.

All tests were two-sided, and statistical significance was defined as a *p* value <0.05. Results are presented as effect estimates with corresponding 95% confidence intervals where appropriate. All statistical testing was performed in SPSS (v28.0.0.0 [190], IBM corp., Armonk, NY, USA).

## 3 Results

A total of 9,522 patients were included in the final analysis. Overall, 9,125 patients (95.8%) provided consent, 184 (1.9%) refused, and 213 (2.2%) had missing or unobtainable consent (Table 1 and Figure 1).

**Table 1:** Cohort characteristics and ambient video consent outcomes.

Characteristic	Total Cohort	Received	Refused	Missing/Unable
Overall cohort	9,522	9,125 (95.8%)	184 (1.9%)	213 (2.2%)
Gender				
Female	4,770	4,560 (95.6%)	99 (2.1%)	111 (2.3%)
Male	4,752	4,565 (96.1%)	85 (1.8%)	102 (2.1%)
Race				
White	7,847	7,541 (96.1%)	136 (1.7%)	170 (2.2%)
Black or African American	1,017	958 (94.2%)	38 (3.7%)	21 (2.1%)
Asian	245	241 (98.4%)	1 (0.4%)	3 (1.2%)
Unknown/Declined/Other	413	385 (93.2%)	9 (2.2%)	19 (4.6%)
Age group, years				
18–44	1,390	1,321 (95.0%)	45 (3.2%)	24 (1.7%)
45–64	2,954	2,830 (95.8%)	66 (2.2%)	58 (2.0%)
65–74	2,542	2,458 (96.7%)	40 (1.6%)	44 (1.7%)
≥75	2,636	2,516 (95.4%)	33 (1.3%)	87 (3.3%)

Data displayed as the number of patients and percentage of consent category. Received denotes documented consent; Missing/Unable denotes absent or incomplete consent capture and is distinct from active refusal.

**Figure 1:**
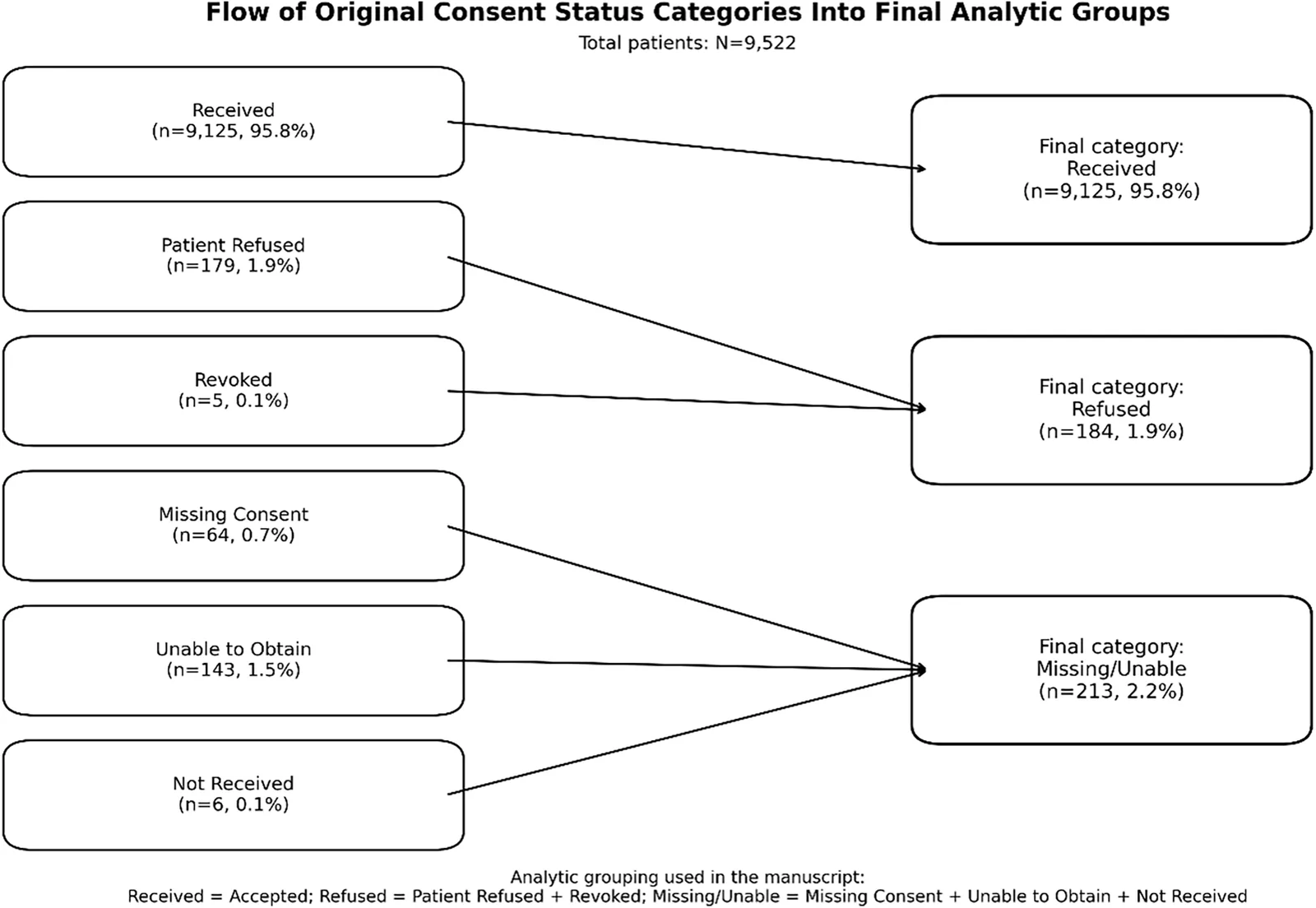
Patient flow through study and derivation of analytic consent status categories. Refused combined patient refused and revoked; missing/unable combined missing consent, unable to obtain, and not received.

Consent acceptance was high across the overall cohort. There was no significant difference by gender. Among female patients, 4,560 of 4,770 (95.6%) consented, compared with 4,565 of 4,752 (96.1%) male patients (*p* = 0.28), and remained unchanged after adjustment for age and race (*p* = 0.30) (Table 2, Panel A).

**Table 2:** Multivariable logistic regression for documented consent acceptance.

Variable	Adjusted OR	95% CI	*p* value
Panel A			
Gender			
Male vs. Female	1.11	0.91–1.36	0.30
Race			
Asian vs. White	2.44	0.90–6.59	0.08
Black or African American vs. White	0.66	0.49–0.88	<0.01
Unknown/Declined/Other vs. White	0.56	0.37–0.83	<0.01
Age			
Per 1-year increase	1.00	0.99–1.01	0.91

Panel B. Secondary model (age categorical)			
Age 18–44 vs. 65–74 years	0.70	0.49–0.95	0.03
Age 45–64 vs. 65–74 years	0.80	0.60–1.06	0.12
Age ≥ 75 vs. 65–74 years	0.70	0.53–0.93	0.01

Panel A is the primary model with age entered continuously. Panel B is the secondary model with age entered categorically; gender and race were included as covariates but are not repeated in Panel B. Reference categories were female, White, and age 65–74 years for Panel B.

aOR, adjusted odds ratio; CI, confidence interval.

Outcome in both models: received consent vs. refused or missing/unable consent.

The three-level consent distribution differed by race and race was also associated with binary documented acceptance. White patients had an acceptance rate of 96.1% (7,541/7,847), Black or African American patients of 94.2% (958/1,017), Asian patients of 98.4% (241/245), and patients categorized as Unknown/Declined/Other of 93.2% (385/413). In primary multivariable model compared with White patients as the reference group, Black or African American patients had lower odds of documented acceptance (adjusted OR 0.66, 95% CI 0.49–0.88, *p* < 0.01), as did patients in the Unknown/Declined/Other category (adjusted OR 0.56, 95% CI 0.37–0.83, *p* < 0.01). The higher observed acceptance among Asian patients was not statistically significant after adjustment (adjusted OR 2.44, 95% CI 0.90–6.59, *p* = 0.08) (Table 2, Panel A; Figure 2).

**Figure 2:**
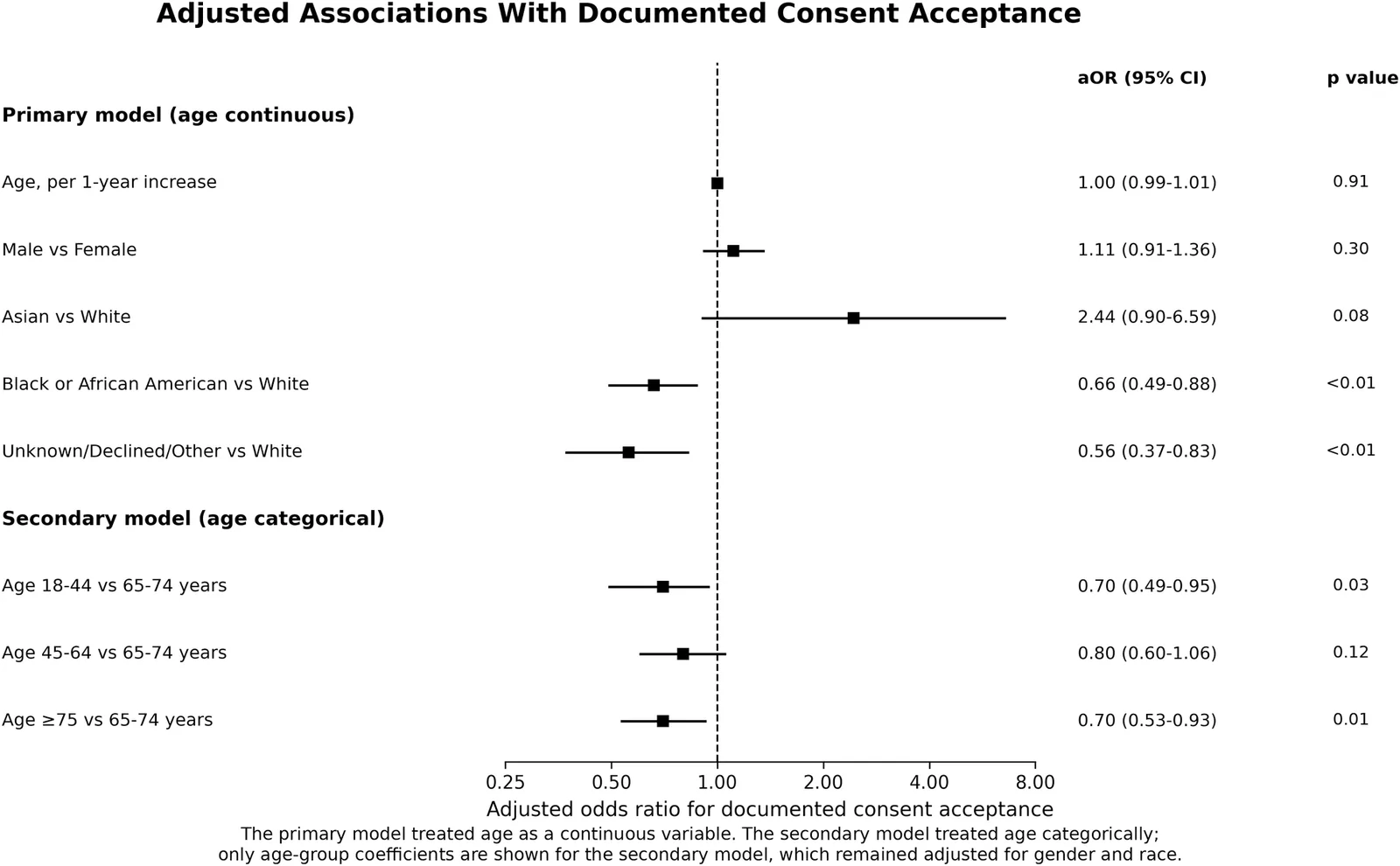
Forest plot showing adjusted associations with documented consent acceptance in primary and secondary binary logistic regression models. The primary model treated age continuously. The secondary model used age categories with 65–74 years as the reference; only age-group coefficients are displayed in that model. All models adjusted for gender and race, and odds ratio greater than 1 indicate higher odds of documented consent receipt.

Documented acceptance was high across all age groups, although modest differences were observed. Among patients aged 18–44 years, 1,321 of 1,390 (95.0%) provided consent, compared with 2,830 of 2,954 (95.8%) among those aged 45–64 years, 2,458 of 2,542 (96.7%) among those aged 65–74 years, and 2,516 of 2,636 (95.4%) among those aged 75 years or older. The three-level consent distribution differed across age groups (*p* < 0.01). The pattern of binary non-acceptance also varied by age. Patients aged 18–44 years had the highest proportion of active refusal (3.2%), while patients aged 75 years or older had the highest proportion of missing or unobtainable consent (3.3%). Patients aged 65–74 years had the highest acceptance and relatively low proportions of both refusal and missing/unable.

In the primary model, continuous age was not independently associated with documented acceptance (adjusted OR 1.00, 95% CI 0.99–1.01, *p* = 0.91) (Table 2, Panel A). In the secondary categorical-age model, with ages 65–74 years as the reference, patients aged 18–44 had lower odds of acceptance (adjusted OR 0.70, 95% CI 0.49–0.95; *p* = 0.03), as did patients aged 75 and older (adjusted OR 0.70, 95% 95% CI 0.53–0.93, *p* = 0.01). The estimate for patients aged 45–64 years was not significant (Table 2, Panel B; Figure 2).

Sensitivity analyses were consistent to alternative categorizations of low-frequency consent statuses. Reclassifying revoked consent as accepted, excluding revoked cases, and restricting the analysis to patients with explicit yes/no decisions did not change the conclusions that documented acceptance was high, gender was not independently associated with acceptance, and Black or African American patients had lower adjusted odds than White patients. The age finding was more sensitive to outcome definition. Continuous age was not associated with acceptance in the primary model, whereas older age was associated with greater odds of acceptance when the analysis was restrcited to explicit yes/no decisions. This pattern indicates that missing or unobtainable consent among older patients influenced the age association.

## 4 Discussion

In this real-world inpatient cohort, documented receipt of consent for ambient monitoring technologies was very high. This finding demonstrates that a standardized operational workflow can achieve high documented uptake in one academic hospital. Consistent with broader literature expected clinical benefit and confidence in human and institutional oversight may contribute to willingness to accept AI-based care, but this present study measured consent documentation rather than patient attitudes (10–12, 14–18).

The high overall acceptance observed here is important because camera-based hospital technologies raise some sensitive issues in digital health implementation. Prior work on video surveillance and inpatient monitoring has emphasized that visual monitoring lives in a distinct ethical space compared with many other digital tools because it directly implicates privacy, dignity, autonomy, and trust (5–7, 9). The acceptance rate remaining above 95% indicates that patients recognized a sufficiently strong clinical or safety rationale, trusted the institution implementing the technology, or found the consent approach acceptable. It may also reflect the pattern seen in AI acceptance literature, in that people often support technology when expected benefits are tangible, but their support depends on whether the technology is framed as assistive, transparent, and governed rather than coercive or opaque (10–12, 14–17, 19). Conversely, agreement during an admission workflow may also reflect social desireability, completion of a routine form, limited attention during acute illness, or concern that refusal could affect care despite written language stating otherwise.

These results are reassuring for implementation because it suggests that consenting for ambient video monitoring, at least in the hospital, is similar between male and female patients. Though age-related findings varied. Acceptance remained high across all age groups, but younger adults were somewhat more likely to actively refuse, whereas the oldest patients were more likely to have missing or unobtainable consent. One possible hypothesis for the higher rate of declination among younger patients is that younger adults may be more digitally fluent yet also more skeptical of passive data collection and surveillance. This finding is also interesting because it differs from much of the broader digital health literature, in which older age is often associated with lower uptake of tools such as telemedicine (36). In the case of ambient video, younger patients may have been more likely to have a digital boundary around privacy and autonomy, whereas older patients may have been less likely to decline outright but more likely to have incomplete or unobtainable consent because of illness, workflow, or communication barriers. Among older inpatients, missing/unable status could reflect acute illness, delirium, cognitive, impairment, communication barriers, timing, or lack of an available surrogate. Importantly, age itself is not equivalent to decisional incapacity; underlying cognitive disorders and the clinical context are more relevant (37, 38). Because it was not possible to identify the exact clinical context, capacity of patients, or exact cognitive assessment in this dataset, this study cannot determine why consent was missing/unable in the older age group. This interpretation is consistent with prior digital health research showing that age can shape technology uptake through multiple pathways, including familiarity with digital systems, health literacy, communication modality, and access barriers (21, 27–29, 31, 32).

An important subgroup finding was the racial difference in documented acceptance. Compared with White patients, Black or African American patients and those categorized as Unknown/Declined/Other had lower adjusted odds. The absolute differences were small, but they can matter when technologies are deployed at scale. These associations should not be interpreted as inherent reluctance or as causal effects of race. Race was an administrative social category, and the models could not adjust for socioeconomic status, education, health literacy, or prior experiences with the health system. The findings may therefore reflect unmeasured structural and relational factors that shape trust, communication, and technology uptake (12, 14–17, 19, 20, 22–26, 30).

Potential mechanisms may explain these racial differences. First, trust in AI and digital surveillance is shaped by prior experiences with healthcare systems, the quality of communication, perceptions of respect, and both historical and ongoing inequities (12, 14–17, 19, 20, 22–26, 30). Second, growing literature has documented bias and unequal performance in healthcare algorithms, which may reasonably heighten concern among groups that have historically been underserved or harmed by health systems (33–35, 37). Third, implementation practices themselves may not be experienced uniformly. Differences in how consent is explained, who presents it, what language is used, and whether privacy protections feel culturally credible may all influence willingness to participate. Literature on cultural safety, culturally competent care, and trust-building suggests that communication, contextual tailoring, representation, and respect for community-specific concerns are essential to equitable adoption of new care models (20, 22–24, 26). Prospective qualitative work is needed before assigning explanations to the observed associations.

From an implementation perspective, our findings argue against a one-size-fits-all rollout strategy. High overall acceptance should not lead institutions to assume that ambient monitoring is socially frictionless. Instead, implementation teams should view subgroup differences as signals for targeted improvement. For example, standardized consent scripts, plain-language educational materials, visible explanation of privacy protections, and clear descriptions of human oversight may help strengthen trust broadly (5–7, 9, 11, 12, 14–19). More tailored efforts may also be needed, including culturally responsive communication, opportunities for questions, and partnership with patient advisors or community representatives from groups that show lower uptake (20, 22–24, 26). In this sense, acceptance analysis becomes an early implementation quality metric, not just a descriptive outcome.

These findings also have implications for governance. The literature on trustworthy AI repeatedly emphasizes that technical validation alone is insufficient; governance must include transparency, accountability, privacy-by-design, fairness assessment, human-centered implementation, and ongoing monitoring after deployment (4, 8, 10, 11, 19, 33, 34, 37). Our study supports that view as a hospital may achieve strong patient acceptance, yet still miss important disparities unless uptake is evaluated at the subgroup level. For ambient video systems, equity monitoring should therefore extend beyond model performance to include consent patterns, revocation patterns, patient concerns, and downstream experience with the technology. This broader approach is especially important because AI systems can deepen disparities not only through biased predictions but also through unequal trust, unequal participation, and unequal access to the potential benefits of digitally enabled care.

The revocation pattern in this study is also informative, as only a very small number of patients withdrew consent after initially agreeing, but this should not be interpreted as proof that initial acceptance was durable. Consent extended to future hospitalizations unless revoked, and low revocation count may reflect satisfaction, but it may unless reflect limited awareness that consent remained active, difficulty locating the revocation pathway, or limited follow-up. Long-term implementation should therefore include simple in-room and post-discharge mechanisms to review or change preferences, periodic affirmation when technology functions or data change, and prospective assessment of post-consent experience.

This study has several limitations. First, it was conducted at one academic hospital with a predominately White cohort. Acceptance patterns may differ across regions, hospital types, patient populations, clinical units, institutional reputations, and ambient technology use cases. Second, the study analyzed operational documentation rather than quality of the consent encounter and process. The high rate could therefore depend on the form, workflow, or institutional perception as much as on the technology itself. Third, cognitive impairment, delirium, acute distress, illness severity, and surrogate involvement were unavailable. Missing/unable status cannot be equated with incapacity, and the validity of individual consent could not be assessed. Fourth, race categories were consolidated for statistical analysis, which may obscure heterogeneity within smaller groups. Finally, documented acceptance did not measure effectiveness, harm, privacy events, patient experience, or durable preference. These limitations preclude causal interpretation and make the the results most applicable as an implementation description rather than a definitive estimate of informed acceptability (15–19, 29–36).

In conclusion, documented consent acceptance for ambient monitoring was high in this academic hospital, with modest differences by race and age-related patterns in refusal versus missing/unable status. The findings support the operational feasibility of obtaining and enforcing documented consent, but they do not establish informed endorsement, or durable preference. Future studies should include measure comprehension and motivations, capture cognitive status and surrogate involvement, test how framing and workflow influence choices, and prospectively evaluate patient experience, revocation, and whether benefits, burdens, and trust are distributed fairly across patient populations.

## Data Availability

The clinical datasets generated and analyzed during this study are not publicly available because they contain protected health information and are subject to Mayo Clinic Institutional Review Board approval and institutional data governance policies. Deidentified derived datasets and analysis code may be made available by the corresponding author upon reasonable request, contingent on institutional review and approval and, where applicable, execution of an appropriate data use agreement. Requests to access the datasets should be directed to helgeson.scott@mayo.edu.
